# From hypoxia single‐cell gene signatures to HIF targeting of AML leukemic stem cells

**DOI:** 10.1002/hem3.59

**Published:** 2024-03-29

**Authors:** Thomas Mercher, Juerg Schwaller

**Affiliations:** ^1^ INSERM U1170, Equipe Labellisée Ligue Contre le Cancer, Gustave Roussy Université Paris‐Saclay Villejuif France; ^2^ Department of Biomedicine, University Children's Hospital beider Basel (UKBB) University of Basel Basel Switzerland

The role of hypoxia and the transcriptional regulation by hypoxia‐inducible factors (HIF) is intensively studied in many tissues and pathologies and gained the most attention in cancer including acute myeloid leukemia (AML).^1^ Here, Velasco‐Hernandez et al. addressed the important issue of the heterogeneity of hypoxia gene signature activation not only between major AML genetic subgroups but also between different populations of patient cells at diagnosis and relapse using single‐cell transcriptomes. Their data support the potential of targeting hypoxia‐induced gene regulation in leukemic stem cells (LSC) in AML.^2^

Oxygen homeostasis is transcriptionally regulated by a family of hypoxia‐inducible factors (HIF) that act as transcriptional activators and repressors. Heterodimeric HIFs are formed by a constitutively expressed HIF1β subunit (encoded by *ARNT*) and a HIF1/2/3α (encoded by *HIF1A, EPAS1, HIF3A*, respectively). While constantly degraded by the proteasome under normoxia, mediated by the oxygen sensor PHD proteins, in hypoxia, HIF‐1/2/3α binds to its cofactor and controls the transcription of target genes in a cell‐context‐specific manner.^3^

In their study, Velasco‐Hernandez et al. first interrogated hypoxia‐regulated gene expression signatures in patient‐derived data sets. They found that the driver genetic lesions determined whether the disease was “HIF^high^,” including cases with core‐binding factor alterations, or “HIF^low^,” including cases with KMT2A rearrangements (KMT2A‐r). They also addressed the HIF expression signature in various leukemic cell populations of 11 patient samples by single‐cell RNA‐sequencing (scRNA) and defined fractions enriched in LSCs (LSC^34^) or not (non‐LSC^34/38^), based on the highest expression of a previously established LSC‐6 expression score and LSC surface markers. Comparison with six different hypoxic gene expression signatures revealed that LSC^34^ cells had the lowest hypoxia score and lowest HIF1A levels in all patients. Importantly, in all genetic AML subgroups, the hypoxia score was higher in LSC^34^ compared to healthy CD34^+^ hematopoietic stem and progenitor cells (HSPCs). Comparing the scRNA signatures from diagnosis and relapse revealed significant inter‐patient transcriptional changes with an overall inverse evolution between hypoxia and the increased LSC‐6 score.

To explore the therapeutic potential of the elevated expression of hypoxia‐related genes including HIF1A in LSC^34^, Velasco and colleagues tested the effects of small‐molecule HIF inhibitors on primary cells from six AML patients. Hypoxic long‐term culture‐initiating cell assays revealed that BAY87‐2243, a molecule reported to inhibit mitochondrial complex I activity impairing hypoxia‐induced HIF‐1α and HIF‐2α upregulation and HIF target genes expression, further reduced the LSC fraction upon combined treatment with Ara‐C associated with reduced expression of selected HIF target genes. Also, they found that the combination of Ara‐C with BAY87‐2243 further reduced the tumor cell burden upon transplantation into NSG mice. Cells from treated mice showed reduced clonogenic potential in vitro and reduced leukemia‐initiating capacity in four out of five AMLs tested. In contrast, the HIF inhibitor did not affect Ara‐C‐mediated reduced engraftment and clonogenic activity of normal HSPCs. Collectively, Velasco et al. unraveled the LSC transcriptomes and particularly hypoxia‐regulated genes at the single‐cell level from AML patients at diagnosis and relapse and provided experimental evidence of synergistic antileukemic activity of small molecule HIF inhibitors and Ara‐C in vitro and in vivo.^2^

With the development of easy‐to‐perform experimental platforms, the number of studies that report AML transcriptomes at the single‐cell level is rapidly increasing.^4^ In their work, Velasco et al. revealed the gene expression signatures of over 100,000 primary blasts from AML patients of the most prevalent genetic subgroups. Therefore, this study represents a major resource to interrogate the molecular trajectories both within the AML cellular hierarchy (e.g., LSC versus non‐LSC) and between diagnosis and relapse. Here, consistent with the observed inverse correlation between the hypoxia signature and cell stemness, most hypoxia targets were more highly expressed in differentiated non‐LSC cells. However, some specific HIF1A targets, including *NPM1* and *CD99*, were significantly higher expressed in LSC^34^ than in non‐LSC^38^ cells. These observations were unexpected as some previous studies reported that HIF1A is mostly expressed in CD34^+^CD38^−^ but not in CD34^+^CD38^+^ cells from AML cells with different genetic driver lesions. The discrepancy may result from the different experimental setups but also from a large number of HIF‐regulated gene targets with messenger RNA expression levels that do not necessarily reflect their biological activity. Further comparisons with datasets combining single nuclei transcriptome and chromatin accessibility could provide additional insights, in particular, whether hypoxia signatures are modulated upon the reported shift toward a B cell‐like program from AML diagnosis to relapse.^5^

Several earlier studies used mouse models to explore the role of HIF for AML induction and maintenance. Upon conditional inactivation of *Hif1a, Hif2a*, or both, Vukovic et al. found that ablation of Hif2a accelerated LSC development but did not affect LSC maintenance in KMT2‐MLLT3‐driven AML. Ablation of *Hif1a* and *Hif2a* also suppressed LSC development driven by HOXA9/MEIS1. However, CRISPR/Cas9‐mediated *HIF2a* ablation did not affect survival, proliferation, and colony formation in normoxia or hypoxia by human KMT2A‐MLLT3^+^ THP1 AML cells. In addition, exposure of KMT2A‐MLLT3^+^ THP1 or NOMO1 cells to BAY87‐224 did not impact the survival under hypoxic conditions.^6^ Others observed that genetic inactivation of *Hif1a* did not impair but rather accelerated the progression of chemotherapy‐exposed KMT2A‐MLLT3‐driven AML raising concerns about HIF targeting for myeloid malignancies.^7^ However, a more recent study found that short hairpin RNA‐mediated knockdown of HIF2α impaired in vitro colony formation and induced myeloid differentiation of multiple AML cell lines in normoxia. HIF2α knockdown also impaired PDX AML progression associated with differentiation in vivo. In addition, a small molecule HIF2α inhibitor (PT2385) induced differentiation of an AML cell line and PDX cells in normoxia. Interestingly, HIF2α seemed to be regulated by retinoid receptors and its inhibition cooperated with ATRA for AML cell differentiation.^8^

The work by Velasco et al. also strongly supports that pharmacological inhibition of hypoxia‐induced HIF activity has antileukemic activity with therapeutic potential.^9^ They found that the antileukemic effects of small‐molecule HIF inhibitors on KMT2A‐MLLT3^+^ AML cells are dependent on synergism with Ara‐C. The mechanisms of the observed synergism of small molecule HIF inhibitors like BAY87‐224 with Ara‐C remain however unclear. Some potential links were provided by an earlier study showing that hypoxia resulted in reduced expression of deoxycytidine kinase, which acts as a cytarabine‐activating enzyme that could be rescued by treatment with BAY87‐2243, suggesting that the synergism primarily increases the anti‐leukemic activity of Ara‐C.^10^

Several strategies are currently explored to selectively interfere with the HIF transcription factors and multiple compounds were reported to inhibit HIF1a activity by various mechanisms including decreasing protein synthesis and/or expression, increasing degradation, interfering with heterodimerization, decreasing DNA binding, or interfering with transcriptional activation. Two of the inhibitors used by Velasco et al. BAY87‐2243 and PX‐478 were both reported to impair HIF1A protein expression but the detailed mechanism remains unknown.^11^ It will be therefore important to further explore the potential synergism of additional HIF inhibitors with standard chemotherapy and to explore the underlying mechanisms.

Although the observations by Velasco et al. appear promising, there are multiple obstacles to overcome toward a clinically efficient HIF‐targeted therapy in AML. First, as indicated by Velasco et al., there are significant differences in HIF gene activation between AML of different genotypes. In some rare cases, this may result from interference with the HIF dimer by a rare AML‐associated ETV6‐ARNT fusion.^12^ Also, as highlighted by the authors and excluded from this study, cases with IDH mutations produce the oncometabolite 2‐hydroxyglutarate, which likely impacts HIF1α stability through inhibition of PHD proteins.^13^ Second, the significant intra‐ and interpatient differences in LSC gene expression signatures between diagnosis and relapse are now starting to be better characterized at the transcriptome and chromatin levels.^14^ Third, due to the complex and wide networks of HIF‐regulated target genes, critical effectors that may provide synergistic strategies will need to be defined. Fourth, despite the large number of compounds that interfere with expression, modification, and transcriptional activation of hypoxia‐regulated HIF factors, we still lack isoform‐selective compounds that specifically switch off the transcriptional activity. Finally, a better understanding of the interplay between hypoxia‐mediated HIF activation consequences and other cellular processes, including mitochondria/metabolism or other transcriptional/epigenetic regulatory mechanisms, will be important.^15^ Indeed, this could result in interesting combinatorial approaches, not only with standard chemotherapy but also with other generally acting antileukemic drugs, such as BH3‐mimetics or DNA methyltransferase inhibitors, or with AML oncogene‐specific drugs (such as menin inhibitors).

Collectively, the work by Velasco et al. gives a deep insight into the AML‐LSC gene expression signatures at the single‐cell level, characterizes hypoxia‐mediated gene expression, and provides proof of concept for the potent synergistic antileukemic activity of HIF inhibition and Ara‐C (Figure 1).

**Figure 1:**
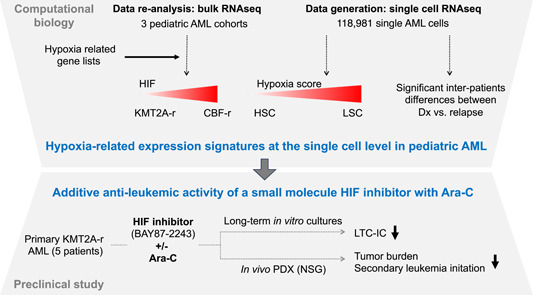
From hypoxic gene expression signatures to antileukemic hypoxia‐inducible factor therapy. AML, acute myeloid leukemia; HSC, hematopoietic stem cell; LSC, leukemic stem cell; LTC‐IC, long‐term culture initiating cell; RNAseq, RNA‐sequencing.

## AUTHOR CONTRIBUTIONS

Juerg Schwaller and Thomas Mercher wrote the manuscript.

## CONFLICT OF INTEREST STATEMENT

The authors have no relevant conflict of interest to declare.

## FUNDING

This research received no funding.
